# The Lipid and Glyceride Profiles of Infant Formula Differ by Manufacturer, Region and Date Sold

**DOI:** 10.3390/nu11051122

**Published:** 2019-05-20

**Authors:** Samuel Furse, Albert Koulman

**Affiliations:** Core Metabolomics and Lipidomics Laboratory, Wellcome Trust-MRL Institute of Metabolic Science, University of Cambridge, Level 4 Addenbrooke’s Treatment Centre, Keith Day Road, Cambridge CB2 0QQ, UK

**Keywords:** Lipidomics, infant nutrition, infant development, lipid metabolism

## Abstract

We tested the hypothesis that the lipid composition of infant formula is consistent between manufacturers, countries and target demographic. We developed techniques to profile the lipid and glyceride fraction of milk and formula in a high throughput fashion. Formula from principal brands in the UK (2017–2019; bovine-, caprine-, soya-based), the Netherlands (2018; bovine-based) and South Africa (2018; bovine-based) were profiled along with fresh British animal and soya milk and skimmed milk powder. We found that the lipid and glyceride composition of infant formula differed by region, manufacturer and date of manufacture. The formulations within some brands, aimed at different target age ranges, differed considerably where others were similar across the range. Soya lecithin and milk lipids had characteristic phospholipid profiles. Particular sources of fat, such as coconut oil, were also easy to distinguish. Docosahexaenoic acid is typically found in triglycerides rather than phospholipids in formula. The variety by region, manufacturer, date of manufacture and sub-type for target demographics lead to an array of lipid profiles in formula. This makes it impossible to predict its molecular profile. Without detailed profile of the formula fed to infants, it is difficult to characterise the relationship between infant nutrition and their growth and development.

## 1. Introduction

There is a mounting body of evidence that the dietary intake of infants has an important role in shaping their lipid metabolism [1,2,3] at a molecular level [4,5,6]. Specifically, there is evidence that the lipid profile in the circulation of formula-fed infants differs considerably to that of breastfed infants by three months of age [6,7,8]. This is consistent with evidence that the milk consumed is associated with the lipid profile in infants [9,10,11,12], children [13] and adults [14,15,16]; as well as with data from a non-human primate that the metabolism of fats and cholesterol is associated with diet after birth [17,18].

This evidence supports the theory of nutritional programming of infants. As self-reporting of mothers across a number of studies indicates that the majority of infants born in the West receive formula (either alone or with breastmilk) [19,20], an understanding of the composition of infant formula is required for investigating the growth and nutritional programming of infants. Despite the wide availability of formula and strict regulations, there is relatively little information beyond the concentrations reported on the package, which only cover macro- and micro-nutrients. A small number of clear and focused studies have profiled formula from particular regions [21,22,23] and even used them in metabolic studies [24,25]. However, most of the studies that focus on the relationship between nutrition and development may only record volume, brand name or type. It is currently unclear whether we can compare infants being fed the same brand or same type, without detailed information on the molecular composition of the formula. This led us to the hypothesis that the lipid composition of formula is consistent between manufacturers, countries or by target demographic.

We therefore embarked upon a detailed survey of commercially available infant formulae in order to uncover the variety between them. A novel high-throughput method for isolating the lipid fraction and a novel three-part method for nanospray-based Direct Infusion Mass Spectrometry (DI-MS) were developed to solve the particular problems of profiling milk in a high throughput manner. The data generated provides evidence of our ability to study the effect of formula in birth cohort studies on growth and development.

## 2. Materials and Methods

*Reagents*—Solvents were purchased from Sigma-Aldrich Ltd. (Dorset, UK) of at least HPLC grade and were not purified further. Lipid standards were purchased from *Avanti Polar lipids* (Alabaster, AL; via Instruchemie, Delfzijl, NL) and used without purification. Consumables were purchased from Sarstedt AG & Co. (Leicester, UK) or Wolf Labs (Wolverhampton, UK).

*Ethics*—This study is intended as an investigation of the lipid and glyceride profile of infant formulae that are commercially available, in order to inform studies of infant metabolism. It is not intended as a critical examination of manufacturer’s methods, choice of ingredients, differences between manufacturing sites etc, or to inform a policy-based comparison with human or other milk. To this end, we have blinded the names of manufacturers, brands and target demographics of products referred to in the main text. However, the identity of these is available in the Supplementary Information in order to facilitate comparison between studies.

*Sample acquisition and preparation*—Formula and fresh milks were purchased from British Supermarkets (J Sainsbury plc, UK; Ocado Retail Ltd., UK) between November 2017 and January 2019. Test samples of Dutch formula were purchased direct through Albert Heijn (NL) and Dirk van den Broek (NL) in November 2017, a full sample set was purchased through Drogist.nl in Spring 2018. South African formula was purchased through Dis-Chem.co.za and shipped to the UK under an import licence (DEFRA, 2018). All formula was stored at −20 °C. Samples were prepared by dispersing formula (100 mg) into water (ddH_2_O, 1 mL) and freeze-thawed once before extraction of the lipid fraction.

*New method: Extraction of the lipid fraction*—We designed experimental procedures that facilitated the profiling of both the phospholipid (PL) and glyceride (triglyceride and diglyceride, TG/DG) fractions in a high throughput manner. This is challenging in milk samples as triglycerides typically dominate (>98%) and have distinct physico-chemical properties to the minor phospholipid component. We therefore developed an existing isolation method for high throughput (384w plate) profiling. The original method was designed for the isolation of both anionic and zwitterionic phospholipids [26,27], to reduce chemical damage during handling [28] and be suitable for isolation of a variety of uncharged lipid species [27] and used here for the same reasons.

*Lipid extraction*—The method used for extracting the lipid fraction was developed from larger-scale methods [26,27] that were designed to better fit the current understanding of lipid chemistry [28] and adapted for high throughput lipid surveys. The solution of formula (40 uL, prepared as above) was injected into a well (96w plate, Esslab Plate+™ (Chromacol, USA), 2·4 mL/well, glass-coated) followed by internal standards (150 µL, Mixture of Internal Standards in methanol (See Supplemental File, Table S1), water (500 µL) and DMT (500 µL, Dichloromethane, methanol and triethylammonium chloride, 3:1:0·005). The mixture was agitated (96 channel pipette) before being centrifuged (3·2× *g*, 2 min). A portion of the organic solution (20 µL) was transferred to a high throughput plate (384w, glass-coated, Esslab Plate+™) before being dried (N_2 (g)_). A second portion of the extract (80 µL) was transferred to a shallow plate (96w, glass-coated) before being dried (N_2 (g)_), washed (hexane, 2 × 80 µL/well) and re-dissolved (DMT, 30 µL). The samples were transferred immediately to the high throughput plate and dried (N_2 (g)_). The dried films were re-dissolved (TBME, 30 µL/well) and diluted with a stock mixture of alcohols and ammonium acetate (100 µL/well; propan-2-ol: methanol, 2:1; CH_3_COO.NH_4_ 7·5 mM). The analytical plate was heat-sealed and run immediately.

*Novel method: Three-part DI-MS for profiling glycerides, phospholipids and fatty acids separately*—In order to overcome the inherent difficulties of profiling the lipid/glyceride fraction of milk samples, we developed DI-MS (used successfully a number of times on human dried blood spots [5,6,7,8], plasma [4,29] and serum [30]) for milk. The abundance of glycerides (98% of signal intensity in positive mode, Figure S1) led us to the hypothesis that they were suppressing the PLs. As triglycerides ionise much less well in negative mode, we explored this mode for profiling phospholipids (Figure S2). This produced evidence for an array of such species, including all major phospholipids. However, we noted that the spectrum contained a considerable number of intensities for triglycerides. Washing the dried lipid film briefly with hexane reduced the signal intensity of triglycerides, increased the number of *m*/*z* signals consistent with phospholipids by around a factor of two and narrowed the variance within phospholipids. Hexane was chosen as although fats readily dissolve in it, its low dielectric constant makes it a poor solvent for lipids. The glyceride profile of the residue was the same as the whole sample and thus this washing step does not appear to be selective between isoforms of triglyceride (Figure S3). We tested for reported lipase activity [31,32,33] in fresh milk using this method. However, the glyceride spectra were dominated by signals of diglycerides that had lost one equivalent of water (DG-H_2_O). These signals consist of both di- and triglycerides that have fragmented under ionisation conditions [4], confounding measurement of the abundance of DGs.

*Data collection & handling*—The DI-MS method used in this study was based on an existing method [30] that measured in both positive and negative ion modes. We added a third section, in which collision-induced dissociation was used in a second negative mode. This additional section focuses on the fatty acid profile of the phospholipid fraction as triglycerides do not ionise well in negative mode and most of them are washed away (hexane wash, Figure S4). Quality control (QC) samples consisted of three formulae (soya-, caprine-, bovine-based, 1:1:1) dispersed in Jersey milk (150 mg/mL) and freeze-thawed once before use.

Positive mode processing used a deviations threshold of 10 ppm and a signal strength threshold of 2. Abundance/Signal intensity was plotted using 25, 50, 100% QC samples and a correlation threshold of 0·75 was used for both whole and petrol-washed samples. Variables with 0% values across all samples were removed before the intensities were signal-corrected. Finally, values more than 4 standard deviations from the average for each variable were considered outliers and discarded. Negative mode processing used a deviations threshold of 10 ppm. QC samples and a correlation threshold of 0·80 was used for both whole and petrol-washed samples. Processing of the negative mode with CID: deviations threshold of 12·5 ppm on a list of fatty acids of chain length 14 to 36 with up to six olefin bonds and/or one hydroxyl group. All signals stronger than noise were carried forward.

**Figure 1:**
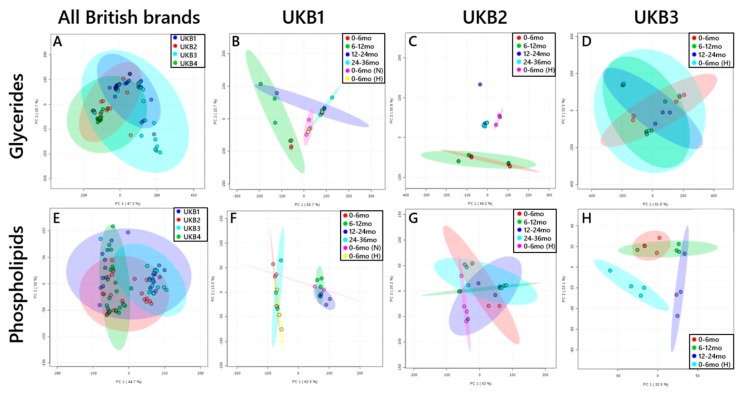
Principal component analyses (PCAs) of infant formulae sold in the UK (2017–2019). Panels (**A–D**), triglycerides and diglycerides: (**A**), All brands; (**B**), British brand 1 (UKB1); British brand 2 (UKB2); British brand (UKB3). Panels E-H, phospholipids: (**E**), All brands; (**F**), British brand 1 (UKB1); (**G**), British brand 2 (UKB2); (**H**), British brand (UKB3). R^2^ values for each component are shown in parentheses on each axis. H, ‘hungry’; N, ‘night’.

**Figure 2:**
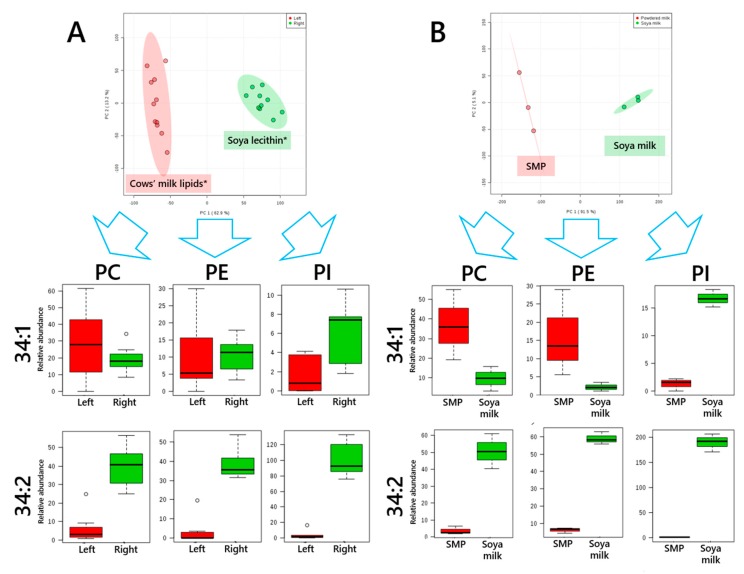
Principal Component Analyses and important loading variables of the phospholipid composition of formula UKB1 and reference materials. Panel (**A**), British brand 1 (UKB1) with subgroups that represent the hypothesised (*) dominant lipid source hypothesised marked (Red, cows’ milk lipids; Green, soya lecithin). (**B**), Plot of lipids from skimmed milk powder (SMP) and soya milk with loadings indicating which variables are the most important for distinguishing the two sample types. PC, phosphatidylcholine; PE, phosphatidylethanolamine; PI, phosphatidylinositol. R^2^ values for each component are shown in parentheses on each axis.

*Statistical Tests*—Univariate statistical tests were carried out using Microsoft Excel 2013 and Principal Component Analyses (PCAs) carried out using Metaboanalyst 4.0 [34]. PCAs were used to identify which samples grouped together in a data-driven manner (e.g., Figure 1). Investigation of the secondary hypothesis that the phospholipid profile comprised soya and/or milk lipids (e.g., Figure 2) used skimmed milk powder and soya milk as comparison samples. Skimmed milk powder is less perishable than fresh animal milk and varies little by season. It therefore represents cows’ milk lipids reliably.

## 3. Results

We began by investigating the variety within the considerable array of infant formula available in the UK. We therefore profiled both the phospholipid and glyceride fractions of infant formulae sold in British supermarkets, 2017-19.

The majority of products available were preparations made from bovine material (typically skimmed cows’ milk), across both ready-made liquid preparations and the larger packages (900 g) used by parents every day. As the latter are expected to provide the bulk of formula-fed infants’ intake, these were investigated in the present study. The lipid signals acquired through positive mode (glycerides, untreated), negative mode (phospholipids, washed with hexane) and fatty acid profile were compared using an unsupervised multi-variate test (Principal Component Analysis, PCA). Samples were compared by brand (British brands 1-4, UKB1-4), date sold (November 2017, June 2018, September 2018 and January 2019) and type (age of infant: 0–6 mo, 6–12 mo, 12–24 mo, 24–36 mo). A full set of PCAs is shown in Figure S5.

These results provided evidence for distinction between brands in glyceride profile (Figure 1A), borne out by the evidence of subgrouping by type in the glyceride plots of UKB1 (Figure 1B) and UKB2 (Figure 1C). UKB3 (Figure 1D) and UKB4 (not shown) did not show subgrouping. The preparations we investigated comprise combinations of palm oil, sunflower oil, rapeseed oil, coconut oil, soya oil, single cell oil, fish oil and oil from *Mortierella alpina*.

There was also evidence for subgrouping by phospholipid composition, with two denser areas in the overall plot (Figure 1E). This distinction is also evident within brands, e.g., UKB1 (Figure 1F) and UKB3 (Figure 1H), suggesting that the composition of products differs between subtypes (target demographic age-range of infant) and date of manufacture. The ingredients listed on the packaging suggested that this distinction may be due to the presence of soya lecithin. PCAs were used to identify the variables that were the most important for distinguishing the two groups in one brand of formula (UKB1, Figure 2A) and which variables best distinguished the lipid composition of skimmed milk powder against soya milk, Figure 2B. Skimmed milk powder and soya milk preparations were used for this comparison as the former contains only milk lipids, where the latter contains no bovine material. The (unsupervised) multivariate analysis (MVA) showed that the same variables were the most important in distinguishing both the different groups of formulae and also cows’ and soya milk (Figure 2B). We used this approach to characterise formula from other regions and from a non-bovine source.

**Figure 3:**
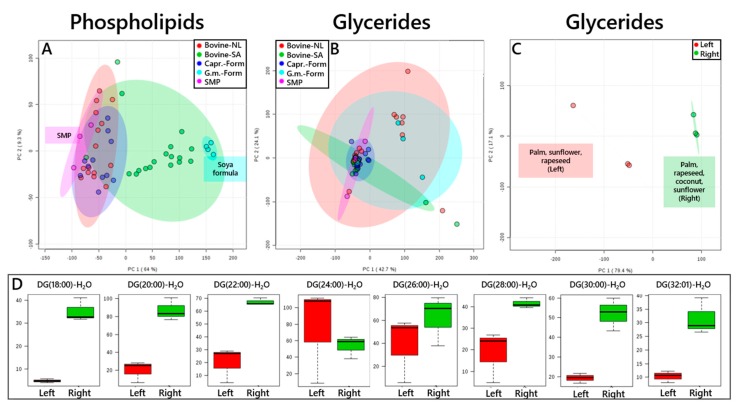
PCAs of bovine-based infant formula sold in South Africa and the Netherlands, caprine- and soya-based infant formulae sold in the UK and skimmed milk powder sold in the UK. Panel (**A**), phospholipids. Panel (**B**), triglycerides and diglycerides. Panel (**C**), Triglycerides and diglycerides for Dutch brand 2 (NLB2), with the three most abundant fatty ingredients marked in two subgroups. Panel (**D**), important loading variables for NLB2 that are consistent with independent profiling of coconut triglycerides. R^2^ values for each component are shown in parentheses on each axis. Capr., caprine; Form, formula; G. m., *Glycine max*; NL, Netherlands; SA, South Africa; SMP, skimmed milk powder.

An unsupervised MVA of the lipid profile of Dutch and South African infant formula based on bovine material and British formula based on caprine material, along with references of skimmed cows’ milk powder and soya-based formula, is shown in Figure 3A. This indicated that the lipid profile of British caprine-based formula differed little from the lipid profile of (cows’) skimmed milk powder, or Dutch infant formula based on cow’s milk. Soya-based formula was appreciably different from skimmed milk powder, indicated by it clustering in PC1, where skimmed milk powder was centred on the opposite side in PC1 (Figure 3A). South African infant formula was scattered between these two points, with around two thirds of these formulae closer to the soya-based formula than skimmed milk powder.

A PCA plot of the glycerides in the same samples (collected in positive ion mode) indicated that the Dutch formula falls into two subgroups (Figure 3C). The ingredients listed on the packaging suggested that the mixture of oils differed in the composition of formula aimed at the younger (0–6 mo, 6–10 mo and 10–12 mo, *viz.* 1, 2 and 3) and older (12–18 mo, 18–24 mo and 24–36 mo, *viz.* 4, 5 and 6) infants (Figure 3C). The ingredients lists of the formulae aimed at younger infants quotes palm, rapeseed, coconut and sunflower oils. The formulae for older infants comprise palm, sunflower and rapeseed oils. This indicates that formulations for younger infants are comprised coconut oil and less sunflower oil than rapeseed oil. The variable loadings that are associated with the results of the unsupervised MVA (Figure 3D) show that the formulae for younger and older infants form two subgroups, albeit with variety within groups (mainly in PC2). These results are consistent with this and data already published on the fatty acid profile of rapeseed, sunflower and coconut oils [35,36]. These studies showed that over 70% of the fatty acids in coconut oil comprised 14 carbons or fewer, where 80% of the fatty acids in sunflower and rapeseed oil comprised 18 carbons or more. The latter two oils differed in that sunflower oil contained about twice as many unsaturated bonds as rapeseed oil. This suggested that the groups of formulations in NLB2 products differ by both the length of the carbon chains of the fatty acid residues and the profile of *mono*- and *di*-unsaturated fatty acid residues.

**Figure 4:**
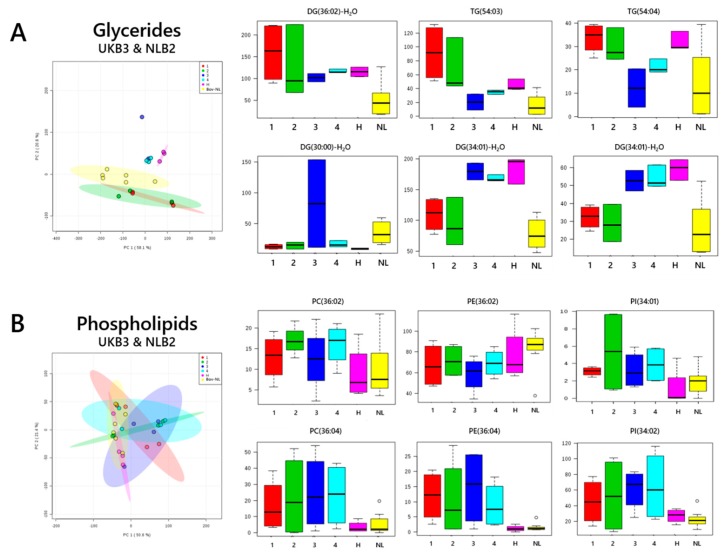
PCAs with important loading variables for Dutch and British brands that are produced by the same manufacturer. Panel (**A**), triglycerides; (**B**), phospholipids. DG, diglycerides; PC, phosphatidylcholine; PE, phosphatidylethanolamine; PI, phosphatidylinositol; TG, triglyceride. 1, 0–6 mo; 2, 6–12 mo; 3, 12–24 mo; 24 mo+; H, 0–6 mo (hungry); NL, Dutch formula.

The variety of lipid and glyceride profiles found in British formula led us to test whether the same formulations were used by one manufacturer across two regions of Western Europe. We used PCAs to compare two brands produced by the same manufacturer, one of which was sold in the UK and the other in the Netherlands. The glyceride fractions of UKB1 grouped clearly (Figure 1C), with some evidence of grouping in the lipid fraction as well (Figure 1G). However, the differences in profile between the subgroups that describe UKB1 subtypes were larger than those between the two subgroups of NLB2 (Figure 4). It was obvious from the ingredients lists precisely how those results might relate to the pattern observed. The difference in the subgroups of UKB1 0–6 mo and 6–12 mo differed by date of manufacture, suggesting that the formulation could have been changed within the order of ingredients listed. The increase in abundance of TG(50:02) in UKB1 formulations (Figure 4A) without a concomitant increase in the abundance of DG-H_2_O(32:01), suggests that there is more FA(18:02) in subtypes 24 mo+ and 0–6 mo (Hungry). This is consistent with a greater proportion of linoleic acid and thus sunflower oil over rapeseed oil.

## 4. Discussion

Most infants in the UK will be exposed to formula in early life [19,20]. For many infants, it will be their main source of nutrition. At the moment, it is unclear if the lipid profile of the formula has an impact on the infant’s growth and development. Our ability to characterise the relationship between the lipid composition of formula and infant growth and development relies entirely on the evidence we have about the composition of formula. In most research, it is assumed that formulae are the same or similar and there is very little appreciation of the possibility that the same brand and type of formula can change abruptly. It is also widely assumed that results of research on the effect of the local formula carried out in a particular region or country will be transferrable to other places.

In this study, we used direct infusion mass spectrometry to survey the lipids and glycerides in an array of British infant formula, which were sourced over a period of 15 months, as well as formula based on cows’ milk sold in the Netherlands and South Africa and fresh animal milk. We developed new methods for extracting the lipid fraction from samples, which facilitated the profiling of both the glycerides and phospholipids and fatty acids in the latter. The triglyceride profiles are difficult to predict from the list of ingredients, because the formula tested comprises as many as seven sources of fat in unknown amounts. All formulae that were profiled comprised up to seven of eight sources of triglycerides (palm oil, rapeseed oil, sunflower oil, coconut oil, fish oil, soya oil, single cell oil, oil from *Mortierella alpina*). Although some of these fats possess a distinct fatty acid profile [35], profiles of the fatty acid composition are not commonly available, may vary between regions and growing seasons or comprise a variety of sources (e.g., single cell oil). The phospholipid fraction of formula is typically constructed of material from milk and/or *Glycine max* (soya), which have contrasting molecular profiles. Because there is a wide range of formulae available and the importance of having a comprehensive overview of the molecular profile increases with the uptake of formula, we needed to develop a high-throughput approach.

The data collected in the present study rely on the methods used to both extract the analytes of interest and survey the mixture of them. The new preparative and analytical methods developed for this study are sympathetic to the experimental challenges that a large set of samples of milk and formula present. For example, there is at least 50 times as much fat as phospholipid in milk. This is an experimental barrier for detailed molecular profiling as the samples comprise species that differ in abundance by more than three orders of magnitude. This difficulty, as well as an interest in the lipid composition of milk, has led to studies focused on the lipid fraction in milk without profiling the glycerides [37,38]. However, we found that using positive mode for the glyceride fraction and negative mode for lipids alleviates many of the problems associated with the suppression of phospholipids by the much more abundant di- and triglycerides. Resolution was improved further by washing phospholipid samples with petroleum ether as this removes the bulk of glycerides. The latter succeeded in increasing the number of phospholipid isoforms measured in negative mode from 153 to 427. The abundance of glycerides means that only a handful were detected in positive mode.

The use of a different mode for profiling phospholipids invites the question of whether or not this is reliable. Despite the possible differences in ionisation efficiency between different lipid head groups in the different modes, the abundance of phospholipids in fresh milk measured using negative mode DI-MS in this study are similar to studies using NMR, an orthogonal technique. ^31^P NMR has been used to profile the relative abundance of phospholipids in cows’ and Ewes’ milk [39], and in formula [38]. Thus, the values reported agree with the abundance of these lipids as measured in negative mode in this study.

However, despite the utility afforded by the present methods, it is not yet known whether other compounds present in milk, that fall below the limit of detection or for which there are not yet standards available, have associated metabolic effects. Our results show that the molecular profile of formula typically deviates significantly from fresh milk, both in terms of glycerides and lipids. However, a recent study of infant formula sold in Spain and human milk from Galician women suggests that in Galicia at least, the overall fatty acid profile of the two is generally similar except for essential poly-unsaturated fatty acids [21]. A combination of the results from that study and the present one therefore suggest that formula may possess different configurations of lipids and fats to human milk, rather than comprising different fatty acids. The essential *poly*-unsaturated fatty acid DHA was only detected in a small fraction of phospholipid isolates from formula in the present study, suggesting that the DHA is invested in triglycerides in infant formula.

It is not clear at present what effect a different presentation of a similar profile of fatty acids in human milk and formula has on infant development and whether it applies universally. There is evidence for different growth trajectories in breast- and formula- fed infants [40,41], for a different lipid profile in the circulation of breast- and formula fed infants [5,6] and for a relationship between the lipid profile of the circulation of infants and the lipid composition of the human milk they are fed [4]. At present, it is not known precisely how much formula and human milk differ in other respects that influence growth, such as calorific value, mineral composition and protein composition. This is partly due to the experimental barriers of determining the variety of dietary intake across the globe but also the relationship between the mother’s diet and lifestyle and the composition of her milk.

The evidence that formulae have contrasting lipid profiles over time and across brands etc. naturally raises the question of how this affects infants. Large-scale metabolic or random controlled trial studies that detail the lipid composition of both formula and the infants that receive it have yet to be reported. However, there are studies of the lipid profile *in circulo* of formula-fed infants. This growing body of work suggests that the profile of lipids in the circulation of formula-fed infants at three months may be just as rich as for age-matched breastfed infants. There is even some evidence that formula-fed infants fall into subgroups, unlike breastfed ones [5,6]. The same studies suggest that the lipid profile of formula-fed infants is less varied at 12 months than that of breastfed ones [6]. Further study may indicate what association, if any, this has with the reported lower risk of obesity in breastfed infants [42]. This invites further detailed studies of nutritional programming.

There is a growing body of evidence that the mother’s diet and lifestyle programmes her infants’ metabolic activity for its lifetime [43,44,45,46,47]. Research in this field has recently broadened to show that the father’s diet can also be influential [48,49]. Although it is not yet clear that the feeding *post partum* has the same magnitude of effects, it is appealing to speculate that different intakes of lipids and fats *post partum* may result in systems programmed differently and thus that behave differently. This may have influenced the development of formulations and led to them more closely resembling the fatty acid profile of breast milk. This may also have contributed to recent changes in the composition of infant formula. However, further work is required to elucidate what effect this has and how best to regulate or inform it.

For example, the British formula we profiled across the time period sampled (November 2017-January 2019) shows a trend of greater use of coconut oil in formula, despite it being considerably more expensive than the most abundant and least expensive fat used, palm oil (coconut oil was 60–250% more expensive than palm oil in the period of 2014–2018 (www.indexmundi.com for prices in Sterling in March 2019). It was already being used in a number of formulae targeted at younger infants (0–6 mo) but during this period it has also appeared in formulae aimed at toddlers. This ingredient is a rich source of triglycerides that comprise fatty acids with chains of 12 or 14 carbons. These closely resemble the triglycerides produced *de novo* in humans.

However, we also note that formula has evolved differently to fresh milk. Formula has been developed to the point of being a powder that readily forms an oil-in-water emulsion in lukewarm or cold water instantaneously, without using agitation equipment. Practical considerations have also led to it being less perishable than fresh milk. The formula we surveyed typically had a use by date of one month from opening and a date of manufacture as much as three months before it was sold. By contrast, cows’ milk is collected twice daily from the teat, stored for up to 48 h before transport to processing centres where it is pasteurised, homogenised and may be skimmed before being sold. Milk therefore typically reaches the supermarket shelf 3–6 days after collection. It has a use by date of around a week from the date of sale and is marked ‘consume within three days of opening’. Furthermore, evidence that fresh milk comprises active lipases [31,32,33] suggests that it will also change during storage in a way that a dry powder similar to those investigated in this study will not. Further work is required to determine the fragility of formula, in particular, the evidence for a considerable number of oxidised triglycerides (~4%). The metabolic effect(s) of species such as oxidised triglycerides have not yet been characterised in infants. There is reasonable evidence to warrant the assumption that these compounds can have possible down-stream biological activity [50,51], for example, though agonism of PPAR receptors by oxidised fatty acids [52].

The suggestion of possible down-stream effects of the components of infant formula, and indeed human milk, naturally raise the question of whether such components should be present in synthetic preparations, and if so, how much. The present study is unable to answer this question formally as it does not comprise metabolic studies. However, further work might include these, perhaps in an animal model, in order to test the relationship between individual molecular components and down-stream cardio-metabolic health.

Lastly, we suggest that the variety of triglyceride and lipid profiles implies that the oil-in-water emulsion structure that formula presumably adopts is flexible to shifts in composition. This suggests that it may be possible to reflect further research about the role of individual lipid or triglyceride components in metabolic health in the composition of formula.

## 5. Conclusions

This study shows that infant formula not only differs by region, manufacturer, source, date of manufacture and target demographic, but moreover that these differences are impossible to predict from the information on the package. A variety of influences shape the lipid and glyceride profile observed, some of which have a dramatic effect on the composition measured. The most pronounced example of this is the use of lecithin from *Glycine max* as an emulsifier.

Our results show that it will be impossible to objectively correlate the effect of lipids and fats in formula with the infant’s growth and development without measuring the actual composition. There is no evidence that results obtained with a particular type of formula of a certain brand in one country can be translated to any other formula or any other country. Detailed metabolic studies, that include profiling of the formula used, are still required to determine how formula-feeding affects infants and their development.

## Supplementary Materials

The following are available online at https://www.mdpi.com/2072-6643/11/5/1122/s1, Figure S1: Profile of the head groups of fresh milk collected in positive mode of Direct Infusion-MS. Panel A, all head groups; B, most abundant types, Figure S2: The glyceride profile of cows’ milk (Bov-) and soya milk, collected in the positive mode of Direct Infusion MS. Whole, unwashed; PW, washed with hexane, Figure S3: Profile of the head groups of fresh milk collected in negative mode of Direct Infusion-MS. Left, whole sample; Right, hexane-washed samples (see methods), Figure S4: Profile of fatty acids collected in negative mode of Direct Infusion-MS with collision-induced dissociation of samples washed with hexane. Left, more abundant species; Right, less abundant species, Figure S5, PCAs of the British formula (UKB1-4) representing glyceride (left column), lipid (middle column) and fatty acid (right column) profiles. Samples are grouped according to Brand (top row), Target demographic (middle row) and Date sold (bottom row), Spreadsheets 1: (unblinded list of infant formulae and manufacturers), Spreadsheets 2: (full signals list), Table S1, Internal lipid, fatty acid, sterol, and triglyceride standards used in the present study.
