# Supplementary material for: The Lipid and Glyceride Profiles of Infant Formula Differ by Manufacturer, Region and Date Sold
Source: Nutrients. 2019 May 20;11(5):1122. doi: 10.3390/nu11051122 (PMC6567151; doi:10.3390/nu11051122)
Supplement: Supplementary file 1 [file nutrients-11-01122-s001.zip › nutrients-505317/Supp Figs/Fig S1.pdf]

Fig. S1

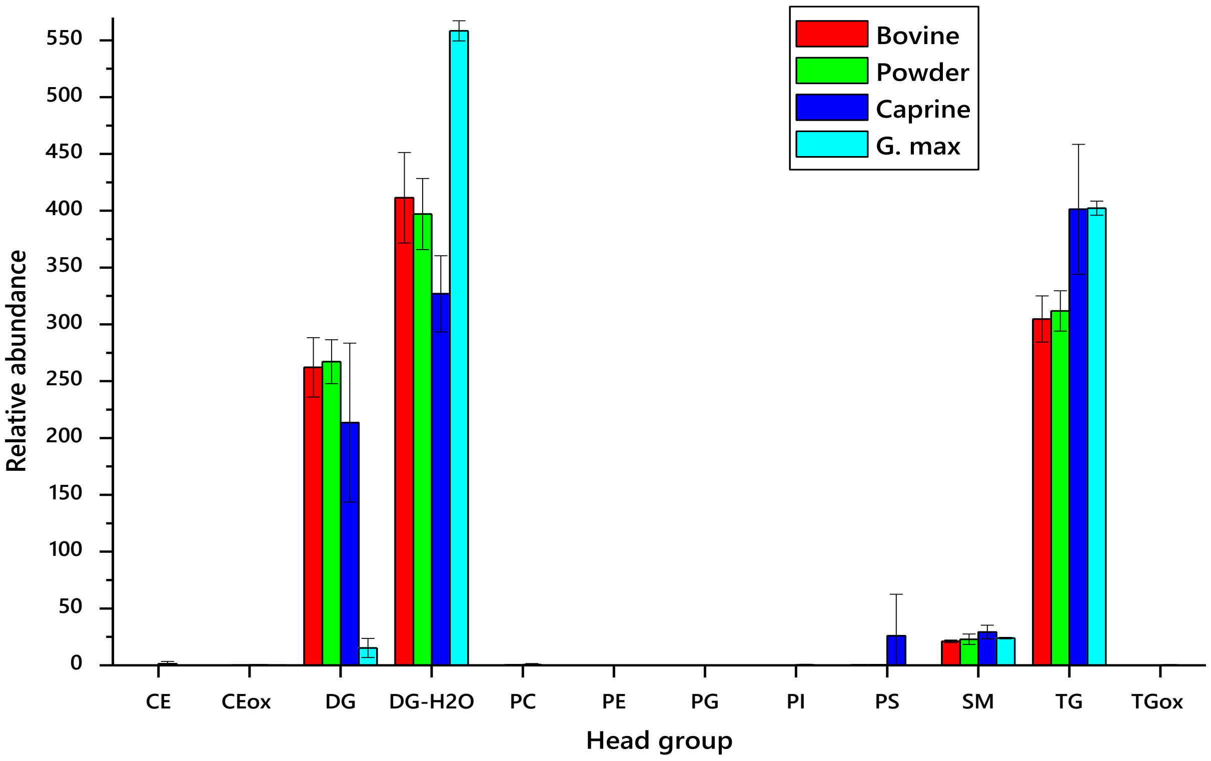

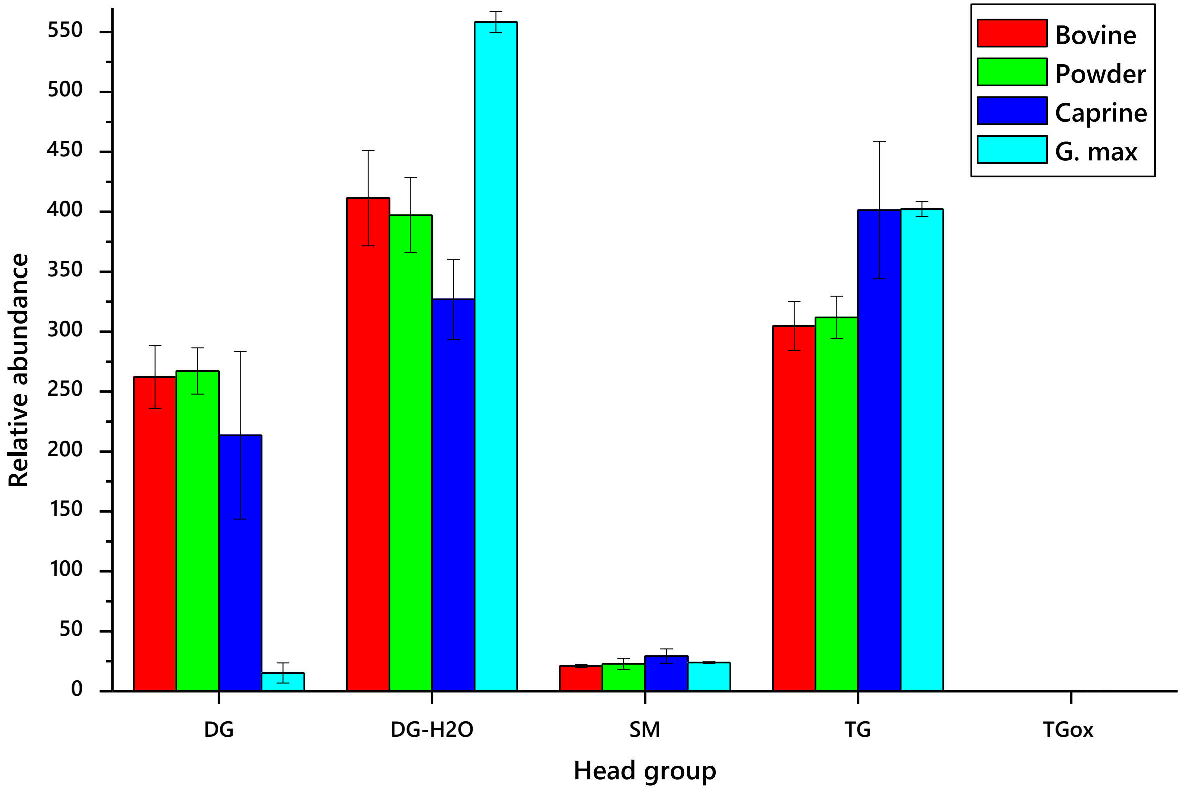

Fig. S1. Profile of the head groups of fresh milk collected in positive mode of Direct Infusion-MS. Panel A, all head groups; B, most abundant types.
