# Supplementary material for: The Lipid and Glyceride Profiles of Infant Formula Differ by Manufacturer, Region and Date Sold
Source: Nutrients. 2019 May 20;11(5):1122. doi: 10.3390/nu11051122 (PMC6567151; doi:10.3390/nu11051122)
Supplement: Supplementary file 1 [file nutrients-11-01122-s001.zip › nutrients-505317/Supp Figs/Fig S2.pdf]

Fig. S2

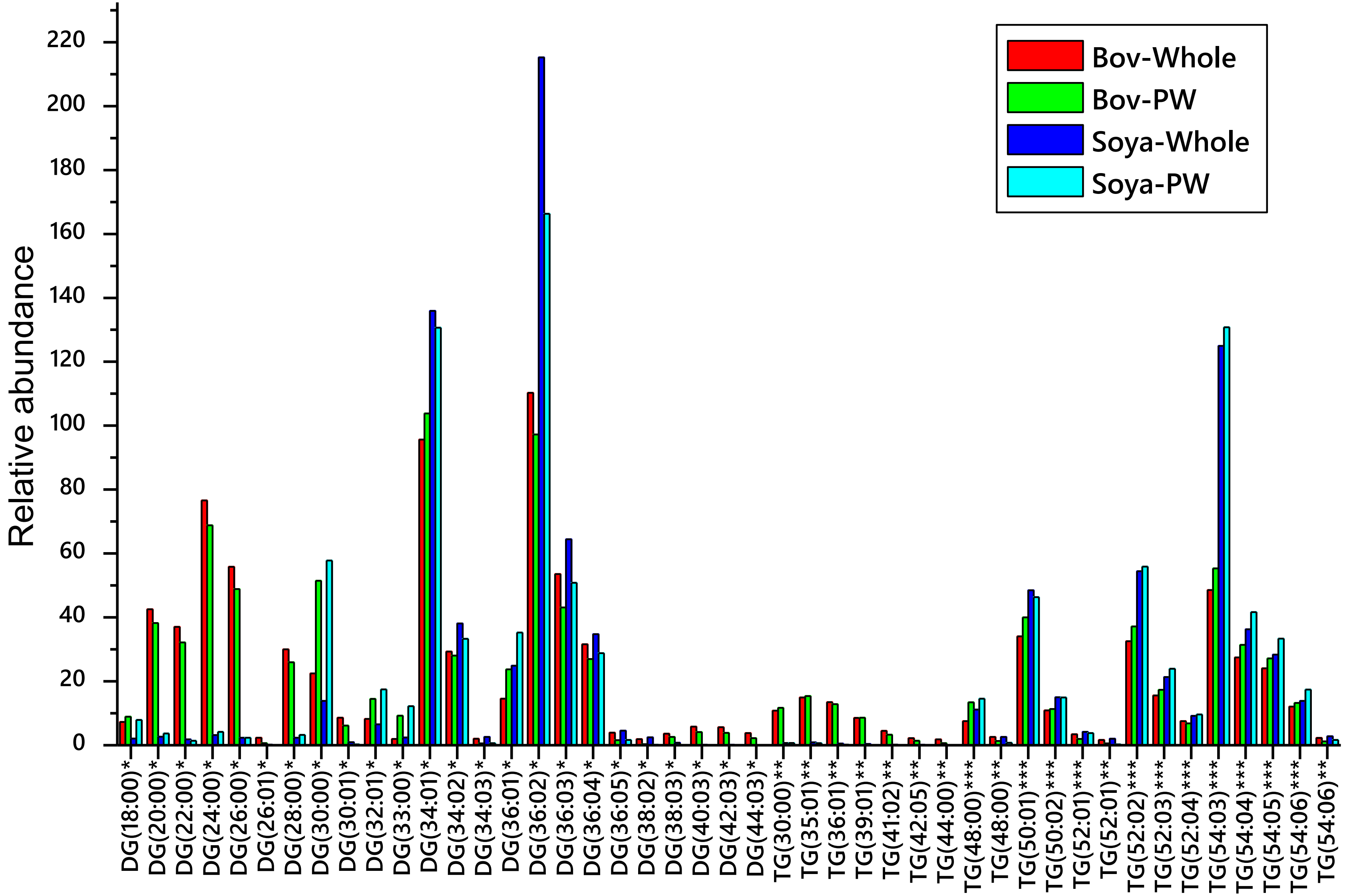

Fig. S2. The glyceride profile of cows' milk (Bov-) and soya milk, collected in the positive mode of Direct Infusion MS. Whole, unwashed; PW, washed with hexane.
