# Supplementary material for: The Lipid and Glyceride Profiles of Infant Formula Differ by Manufacturer, Region and Date Sold
Source: Nutrients. 2019 May 20;11(5):1122. doi: 10.3390/nu11051122 (PMC6567151; doi:10.3390/nu11051122)
Supplement: Supplementary file 1 [file nutrients-11-01122-s001.zip › nutrients-505317/Supp Figs/Fig S4.pdf]

Fig. S4

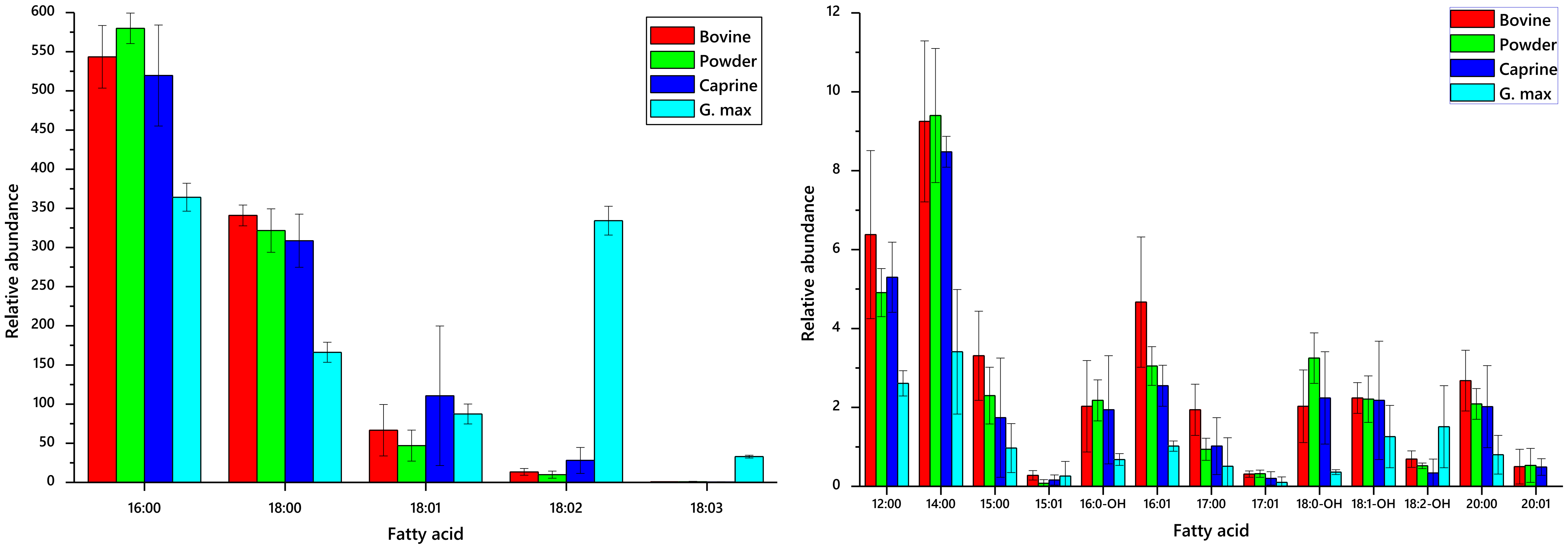

Fig. S4. Profile of fatty acids collected in negative mode of Direct Infusion-MS with collision-induced dissociation of samples washed with hexane. Left, more abundant species; Right, less abundant species.
