# Supplementary material for: The Lipid and Glyceride Profiles of Infant Formula Differ by Manufacturer, Region and Date Sold
Source: Nutrients. 2019 May 20;11(5):1122. doi: 10.3390/nu11051122 (PMC6567151; doi:10.3390/nu11051122)
Supplement: Supplementary file 1 [file nutrients-11-01122-s001.zip › nutrients-505317/Supp Figs/Fig S5.pdf]

Fig S5

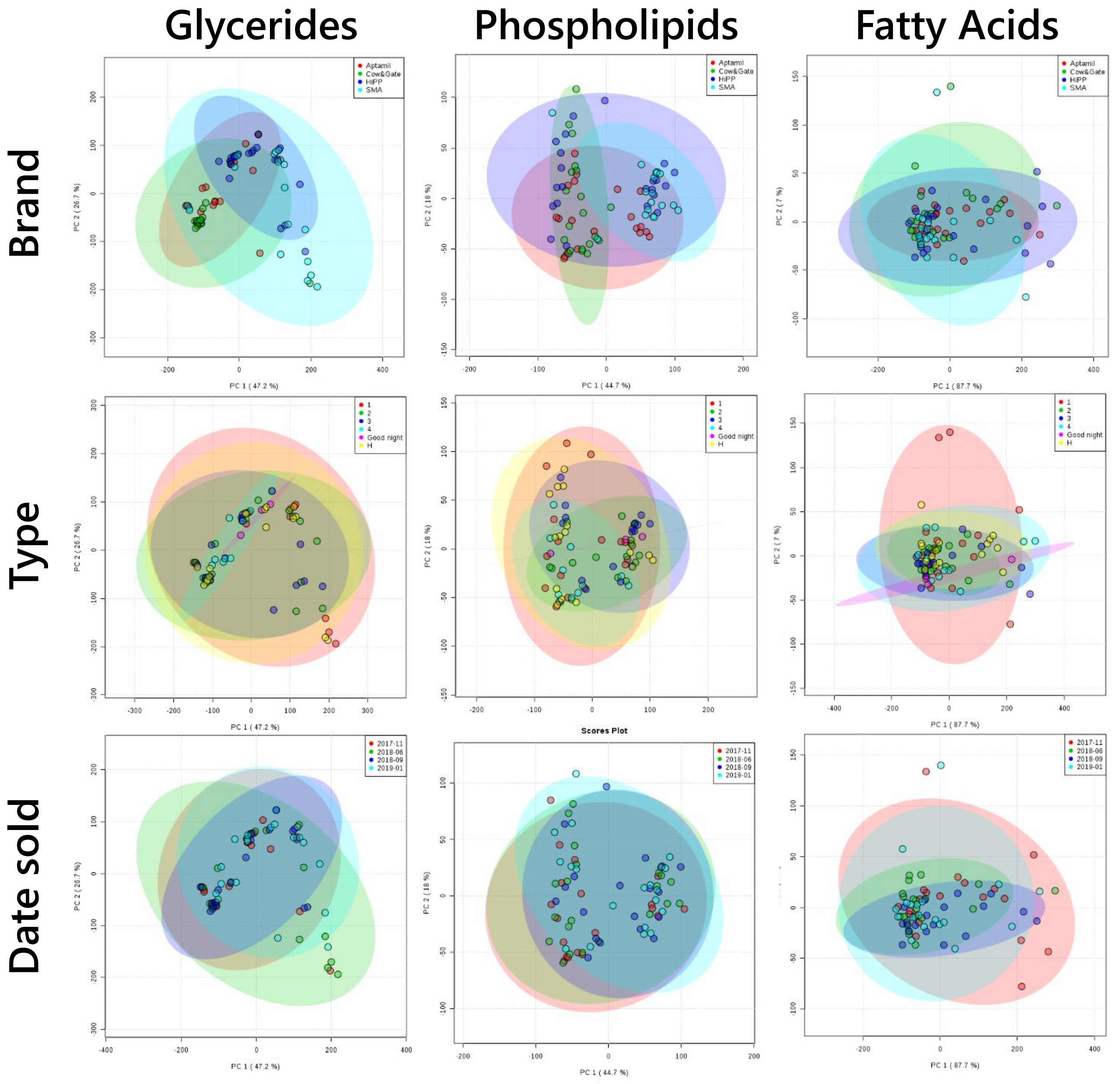

Fig. S5. PCAs of the British formula (UKB1-4) representing glyceride (left column), lipid (middle column) and fatty acid (right column) profiles. Samples are grouped according to Brand (top row), Target demographic (middle row), and Date soled (bottom row).
