# Supplementary material for: The Lipid and Glyceride Profiles of Infant Formula Differ by Manufacturer, Region and Date Sold
Source: Nutrients. 2019 May 20;11(5):1122. doi: 10.3390/nu11051122 (PMC6567151; doi:10.3390/nu11051122)
Supplement: Supplementary file 1 [file nutrients-11-01122-s001.zip › nutrients-505317/Supp Figs/Fig S5.pptx]

## Slide 1
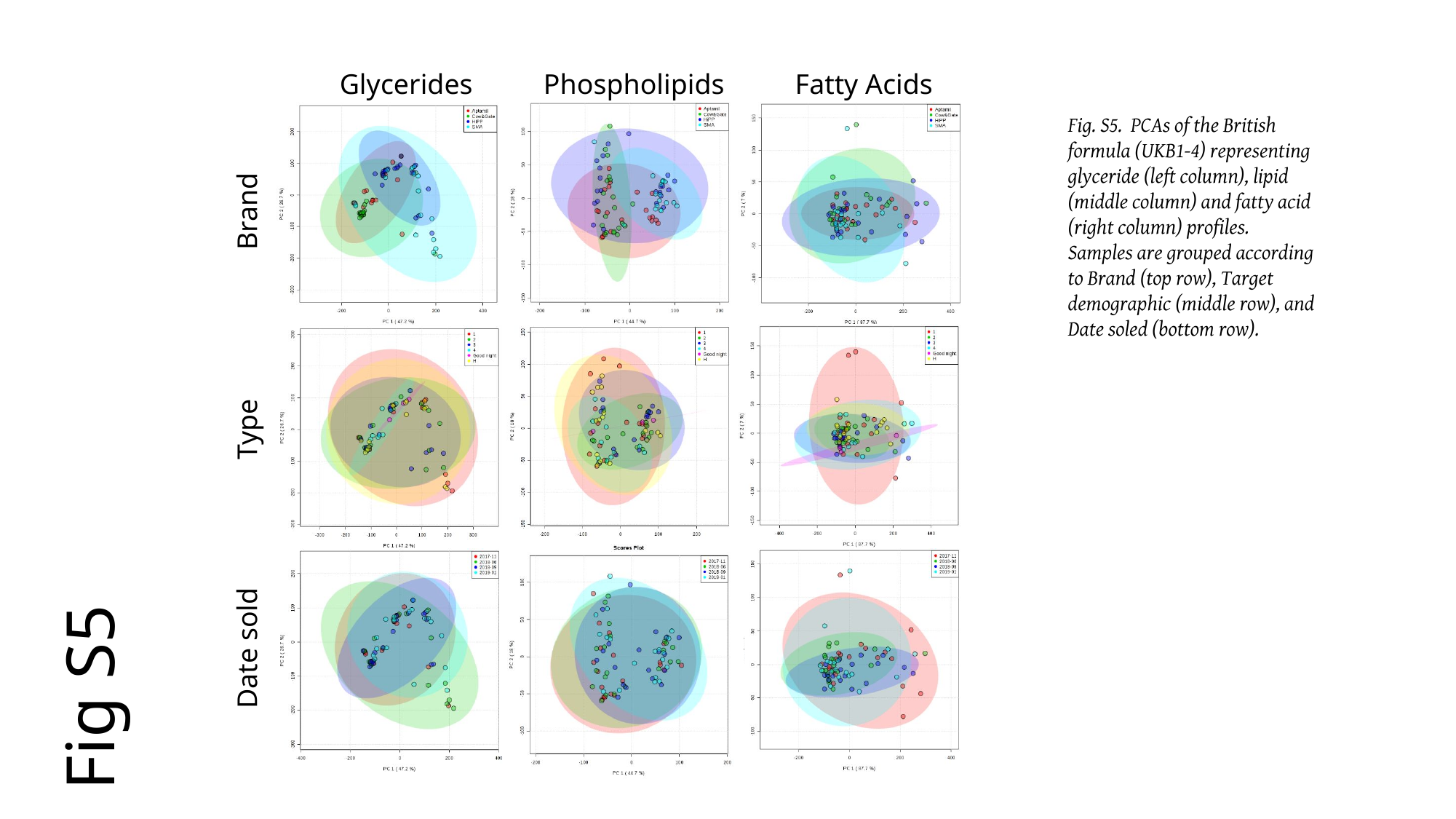

Glycerides Phospholipids Fatty Acids
# Fig S5
Fig. S5. PCAs of the British formula (UKB1-4) representing glyceride (left column), lipid (middle column) and fatty acid (right column) profiles. Samples are grouped according to Brand (top row), Target demographic (middle row), and Date soled (bottom row).
Date sold Type Brand
