# Supplementary material for: The Lipid and Glyceride Profiles of Infant Formula Differ by Manufacturer, Region and Date Sold
Source: Nutrients. 2019 May 20;11(5):1122. doi: 10.3390/nu11051122 (PMC6567151; doi:10.3390/nu11051122)
Supplement: Supplementary file 1 [file nutrients-11-01122-s001.zip › nutrients-505317/Supp Figs/Table S1.pptx]

## Slide 1
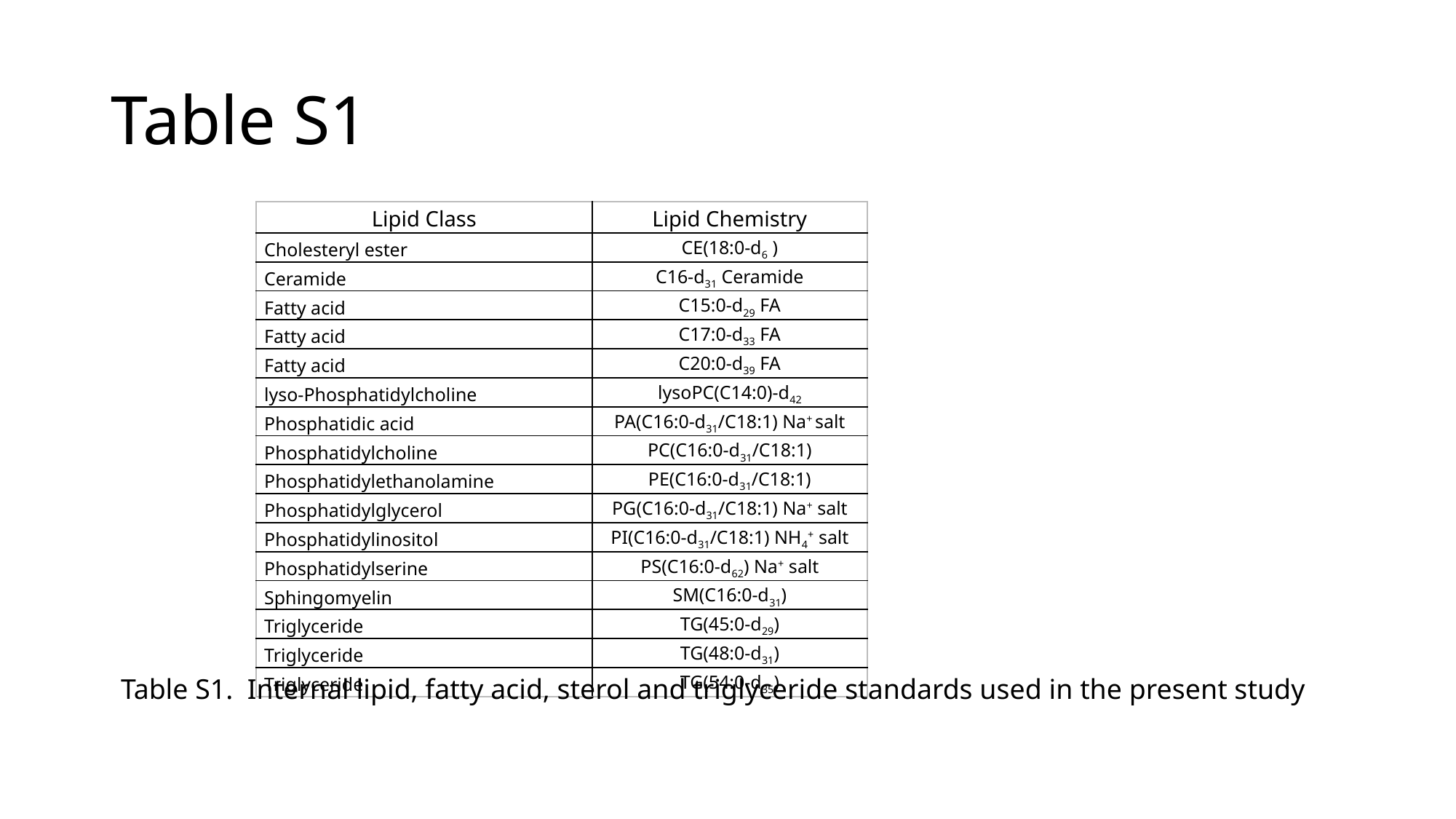

# Table S1
| Lipid Class | Lipid Chemistry |
| --- | --- |
| Cholesteryl ester | CE(18:0-d6 ) |
| Ceramide | C16-d31 Ceramide |
| Fatty acid | C15:0-d29 FA |
| Fatty acid | C17:0-d33 FA |
| Fatty acid | C20:0-d39 FA |
| lyso-Phosphatidylcholine | lysoPC(C14:0)-d42 |
| Phosphatidic acid | PA(C16:0-d31/C18:1) Na+ salt |
| Phosphatidylcholine | PC(C16:0-d31/C18:1) |
| Phosphatidylethanolamine | PE(C16:0-d31/C18:1) |
| Phosphatidylglycerol | PG(C16:0-d31/C18:1) Na+ salt |
| Phosphatidylinositol | PI(C16:0-d31/C18:1) NH4+ salt |
| Phosphatidylserine | PS(C16:0-d62) Na+ salt |
| Sphingomyelin | SM(C16:0-d31) |
| Triglyceride | TG(45:0-d29) |
| Triglyceride | TG(48:0-d31) |
| Triglyceride | TG(54:0-d35) |
Table S1. Internal lipid, fatty acid, sterol and triglyceride standards used in the present study
